# Association of Metabolic Syndrome Components and Nutritional Status with Kidney Cancer in Young Adult Population: A Nationwide Population-Based Cohort Study in Korea

**DOI:** 10.3390/biomedicines11051425

**Published:** 2023-05-11

**Authors:** Hee Yeon Lee, Kyung-Do Han, In Sook Woo, Hyuk-Sang Kwon

**Affiliations:** 1 Division of Oncology, Department of Internal Medicine, Yeouido St. Mary’s Hospital, College of Medicine, The Catholic University of Korea, Seoul 07345, Republic of Korea; 2 Department of Statistics and Actuarial Science, Soongsil University, Seoul 06978, Republic of Korea; 3 Division of Endocrinology and Metabolism, Department of Internal Medicine, Yeouido St. Mary’s Hospital, College of Medicine, The Catholic University of Korea, Seoul 07345, Republic of Korea

**Keywords:** kidney cancer, metabolic syndrome, age, young adults

## Abstract

The aim of this study was to determine the association of metabolic syndrome (MetS) with kidney cancer and the impact of age and gender on such an association. Using the Korean National Health Insurance Service database, 9,932,670 subjects who had check-ups in 2009 were followed up until the diagnosis of kidney cancer or death or until 2019. Kidney cancer was significantly associated with MetS (HR 1.56). This association was higher in the younger age group (HR: 1.82, 1.5, and 1.37 in 20–39 years, 40–64 years, and ≥65 years, respectively). In terms of the association of kidney cancer with obesity and central obesity, young-aged males showed higher HR for kidney cancer than old-aged ones (HR of obesity: 1.96, 1.52, and 1.25; HR of central obesity: 1.94, 1.53, and 1.3 in 20–39 years, 40–64 years, ≥65 years, respectively), while young-aged females showed lower HR. Kidney cancer was associated with obesity and MetS. The association was higher in younger populations than in older ones. Regarding gender, MetS, obesity, and central obesity showed higher associations with kidney cancer in younger aged male population, while there was no significant difference in such associations according to age in the female population.

## 1. Introduction

Globally, kidney cancer accounts for more than 342,000 incident cases and 131,000 deaths each year [1]. It is the 14th most common cancer and the 15th most common cause of cancer death. Its incidence is showing an upward trend [2]. The annual estimated cost of metastatic kidney cancer is about $1.6 billion [3]. It is increasing due to new emerging therapeutics. Considering this large economic burden, kidney cancer is one of the most important cancers. It is known to be associated with high body mass index (BMI), smoking, hypertension (HTN), and chronic kidney disease [4]. A meta-analysis evaluating 18 prospective studies has reported a positive association between kidney cancer risk and HTN [5]. Obesity and smoking can increase the incidence and mortality of kidney cancer. These factors have more effects on young subjects than on older subjects [4,6]. The incidence and mortality of kidney cancer show significant regional variations. Kidney cancer ranks high in developed countries [7]. Its incidence is twofold higher in males than in females.

Metabolic syndrome (MetS) is a cluster of conditions including central obesity, diabetes, HTN, decreased high-density lipoprotein (HDL), and elevated triglyceride (TG) levels. MetS causes a low-grade chronic inflammatory state and insulin resistance, resulting in increased risks of coronary heart disease, stroke, and other vascular diseases. Many studies have reported that MetS can increase cancer risk, and it has been suggested that an inflammatory state and insulin resistance are the pathophysiologies involved in such an increased risk [8,9,10,11]. Due to the huge economic burden of kidney cancer, for intensified public health initiatives, a systemic analysis using public big data is needed regarding its modifiable risk factors, including smoking and obesity, with a larger impact on young subjects than on older subjects, and its regional variations. In our study, we evaluated the association between MetS and the risk of kidney cancer with age using data from approximately 10 million adults from the Korean National Health Insurance Service (NHIS) database. We further assessed whether there was a sex difference in the relationship between MetS and kidney cancer.

## 2. Methods

### 2.1. Data Source

The government of South Korea provides regular biennial health check-ups to the public and manages the Korean NHIS database [12]. Since 2009, 15 million people (about 70% of the total population) have been screened in the NHIS. From NHIS data, extensive patient-related data, including patient demographics, results of nHealth screening (height, weight, waist circumference, blood pressure, the status of alcohol consumption, smoking, and exercise, and laboratory examinations after overnight fasting), and diagnostic codes according to the International Classification of Diseases, 10th Revision, Clinical Modification (ICD-10-CM) were retrieved.

### 2.2. Study Population

**Figure 1:**
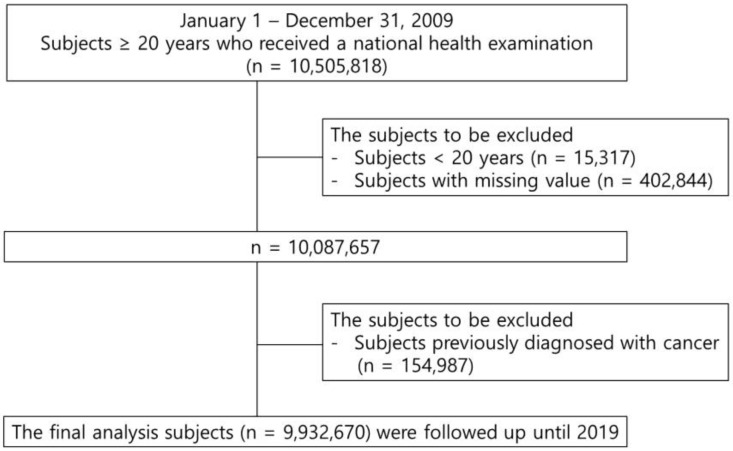
Study Design.

A total of 9,932,670 subjects aged ≥ 20 years with check-ups between January 2009 and December 2009 who had no history of any malignancy or missing data were included. Figure 1 shows the study design. They were followed up until the diagnosis of kidney cancer or death, or until December 2019. This study was conducted according to the Declaration of Helsinki. It was approved by the Institutional Review Board of the Catholic University of Korea (No.SC20ZESI0143). Informed consent was waived because only anonymized and deidentified data were used. This study followed Reporting studies Conducted using Observational Routinely collected health Data (RECORD) guidelines.

### 2.3. Definition

Cases with kidney cancer were defined as having an ICD-10 code of C64, and cases with other “C” codes were excluded. Self-reported lifestyle variables including smoking status (none, ex-smoker, or current smoker) and the intensity of regular exercise (low [<3 times of vigorous intensity or <5 times of moderate intensity/week] or high [≥3 times of vigorous intensity or ≥5 times of moderate intensity/week]).

BMI was defined as weight/height^2^ (kg/m^2^), and obesity was defined as BMI ≥ 25 kg/m^2^. Central obesity was defined as a waist circumference (WC) ≥ 90 cm for men and ≥80 cm for women according to Asian standards [13]. Elevated blood pressure was defined as systolic/diastolic blood pressure (SBP/DBP) ≥ 130/85 mmHg or a prescription history of an antihypertensive agent under ICD10-CM codes I10-13 and I15 (at least one insurance claim per year).

After overnight fasting over 8 h, blood sampling was conducted, and serum levels of glucose, total cholesterol, triglycerides (TG), high-density lipoprotein cholesterol (HDL), and low-density lipoprotein cholesterol (LDL) were assessed. Elevated fasting plasma glucose level was defined as a fasting plasma glucose level ≥ 100 mg/dL or a prescription history of an antidiabetic agent under ICD10-CM codes E11-E14. The definition of MetS followed the Association/National Heart, Lung, and Blood Institute (AHA/NHLBI) criteria for cases with ≥3 of the following five components: abdominal obesity, elevated blood pressure, elevated fasting glucose, elevated TG (≥150 mg/dL or on drug treatment for elevated TG), and reduced HDL (<40 mg/dL for men, <50 mg/dL for women, or on drug treatment for reduced HDL).

### 2.4. Statistical Analyses

The incidence of kidney cancer was calculated as the “number of events/person-time at risk”. Renal duration (person-year) was the period between the date of the health check-up and the date of the diagnosis of kidney cancer, the date of death, or the date of the last follow-up (31 December 2015). To evaluate the statistical difference in baseline characteristics, the Pearson χ^2^ test was used. A Cox proportional hazards model was used to assess the hazard ratio (HR), 95% confidence interval (CI), and effects of MetS on kidney cancer according to age. Adjustment was done for potential confounding factors such as age, sex, smoking status, and physical activity. Younden’s index was used to evaluate the association of the cut-off levels of WC and BMI with kidney cancer risk. All statistical analyses were carried out using SAS version 9.4 (SAS Institute, Cary, NC, USA) and R software version 3.6.0 (The R Foundation for Statistical Computing, Vienna, Austria, http://www.Rproject.org, accessed on 10 November 2022). Statistical significance was considered when the two-sided *p*-value was less than 0.05.

## 3. Results

### 3.1. Baseline Characteristics of Study Population

A total of 9,932,670 subjects were included. Kidney cancer was diagnosed in 12,758 subjects. The mean renal duration was 4.49 person-years in the population with kidney cancer and 8.26 person-years in the population without kidney cancer. Table 1 shows the baseline characteristics of subjects with or without kidney cancer according to age. BMI, WC, SBP, and TG were higher while HDL was lower in the population with kidney cancer than in the population without kidney cancer, regardless of age. Glucose and DBP were higher in the group with kidney cancer except for those in old age (≥65 years). The value gap of MetS components (WC, BP, glucose, HDL, and TG) between the population with kidney cancer and the population without kidney cancer showed a tendency to be dominant in the young age group (20–39 years).

### 3.2. Association of Metabolic Syndrome with Kidney Cancer According to Age

Table 2 and Table 3 show the association of MetS with kidney cancer according to age. Model 1 was a non-adjusted model. Model 2 was a model adjusted for smoking, alcohol consumption, and physical activity. BMI and WC were divided into five groups. The HR for kidney cancer was higher with increasing BMI and WC (Table 2). Conversely, low BMI (<18.5) and WC (<70 cm in males, <65cm in females) showed decreased risk of kidney cancer (HRs [95% CIs]: 0.78 [0.68–0.9] for low BMI and 0.7 [0.61–0.81] for low WC). Regardless of age, every metabolic component showed a higher HR for kidney cancer in both models 1 and 2 (Table 3). Those with MetS showed a significantly higher risk for kidney cancer (HR [95% CIs]: 1.56 [1.51–1.62]). The risk for kidney cancer was higher in younger age groups (HRs [95% Cis]: 1.82 [1.6–2.07], 1.5 [1.43–1.57], and 1.37 [1.29–1.47] for 20–39 years, 40–64 years, and ≥65 years, respectively). More MetS components were related to higher HR for kidney cancer, and HR was higher in younger age groups (Table 3). After grouping subjects by the presence of obesity and MetS, subjects with both obesity and MetS had a higher HR for kidney cancer than subjects who had either obesity or MetS (Table 3). The younger age group (20–39 years) had a higher HR for kidney cancer than the older age group, regardless of the presence of obesity or MetS (Table 3). The cutoff levels of BMI and WC for kidney cancer risk showed a tendency towards decreasing cutoff levels in the younger population (Table 4).

### 3.3. Gender Difference in the Association of Kidney Cancer with Metabolic Syndrome

An analysis according to gender was done. BMI and WC were divided into five levels (Table 5). In the same age group, those with a higher BMI or WC showed a higher HR for kidney cancer in both males and females, except young (20–39 years) females. Those with MetS showed a higher HR for kidney cancer in the young-aged group, regardless of gender. In 20–39 years, 40–64 years, and ≥65 years, HRs (95% CIs) for kidney cancer in males were 1.82 (1.6–2.09), 1.55 (1.41–1.63), and 1.42 (1.15–1.36), respectively, and HRs (95% CIs) for kidney cancer in females were 1.61 (0.91–2.83), 1.39 (1.27–1.52), and 1.31 (1.15–1.46), respectively. In terms of obesity and central obesity, young-aged males showed higher HR for kidney cancer than old-aged males (in 20–39 years, 40–64 years, and ≥65 years, HRs [95% CIs] for kidney cancer in those with obesity: 1.96 [1.74–2.21], 1.52 [1.44–1.6], and 1.25 [1.15–1.36], respectively; HRs for kidney cancer in those with central obesity: 1.94 [1.7–2.21], 1.53 [1.44–1.62], and 1.3 [1.2–1.41], respectively), whereas female showed lower HR for kidney cancer in the young-aged group (in 20–39 years, 40–64 years, and ≥65 years, HRs [95% CIs] for kidney cancer in those with obesity: 1.41 [0.98–2.03], 1.41 [1.3–1.51], and 1.29 [1.15–1.45], respectively; HRs for kidney cancer in those with central obesity: 1.21 [0.72–2.01], 1.33 [1.21–1.47] and 1.25 [1.11–1.4], respectively). In young-aged females, glucose, HDL, and TG showed higher HR for kidney cancer (in 20–39 years vs. in all age groups: HRs [95% CIs] of elevated fasting glucose, 1.38 [0.96–1.98] vs. 1.24 [1.15–1.32]; low HDL, 1.35 [0.99–1.83] vs. 1.29 [1.21–1.38]; high TG, 1.52 [1.01–2.3] vs. 1.25 [1.17–1.34]). However, in young-aged males, those components did not significantly increase the HR for kidney cancer (in 20–39 years vs. in all age groups: HRs [95% CIs] of elevated fasting glucose, 1.08 [0.95–1.24] vs. 1.21 [1.16–1.26]; low HDL, 1.39 [1.2–1.6] vs. 1.43 [1.37–1.49]; high TG, 1.27 [1.13–1.44] vs. 1.33 [1.27–1.38]). MetS-only populations without obesity showed higher HR for kidney cancer in younger groups regardless of age. Young-aged males had a higher HR for kidney cancer regardless of whether they only had MetS (HRs [95% CIs] in 20–39 years vs. all age groups: 1.53 [1.15–2.04] vs. 1.49 [1.4–1.59]), obesity (HRs [95% CIs]: 1.79 [1.55–2.06] vs. 1.34 [1.26–1.42]), or both MetS and obesity (HRs [95% CIs]: 2.49 [2.13–2.91] vs. 1.97 [1.88–2.08]). In young-aged females, MetS, but not obesity, significantly increases the risk of kidney cancer (HR: 2.38, 95% CI: 1.05–5.37).

## 4. Discussion

This study aimed to determine the association of MetS with kidney cancer and the impact of age and gender on such an association using the NHIS database. Overall, the population with kidney cancer had higher BMI, WC, glucose, BP, and TG but, lower HDL than the population without kidney cancer (Table 1). BMI and WC showed positive associations with kidney cancer (Table 2). MetS showed a higher association with kidney cancer in the young-aged population (20–39 years) than in other populations (Table 3). The young-aged group showed a closer association of kidney cancer with obesity, central obesity, low HDL, and high TG than other age groups. MetS without obesity showed a positive association with kidney cancer (Table 3). This association was higher in the young-aged group than in other age groups (Table 3). Younden’s index is commonly used to evaluate the accuracy of diagnostic tests. In this study, it was used to assess the impact of decreasing cutoff levels of BMI and WC in the young adult population (Table 4). The increasing cutoff levels with age highlight the importance of overweight and obesity for young adults compared to older ones as a risk factor for kidney cancer. Regarding gender, MetS, obesity, and central obesity showed significant associations with kidney cancer in young-aged males but not in young-aged females (Table 5).

The association of obesity with kidney cancer is well known. The association between obesity and renal cell cancer (RCC) has been reported in a large prospective US cohort [4]. BMI and the incidence of RCC were positively correlated. A case-control study including 271 RCC cases in China has reported the association of obesity with RCC in Chinese men but not in Chinese women [14]. The association of kidney cancer with obesity was also found in the present study (HR 1.48, model 2).

HTN has been reported to be associated with an increased risk of kidney cancer [15]. A meta-analysis including 18 prospective studies has reported a positive association between HTN and kidney cancer [15]. The history of HTN was related to a 67% increased risk of kidney cancer, and a 10-mmHg increase in SBP and DBP was linked to 10% and 22% increased risks of kidney cancer, respectively [5]. A case-control study including 271 RCC cases in China has reported an independent association of HTN with kidney cancer [14]. A nationwide population-based cohort study in Korea has reported that the risk of kidney cancer is increased with BP in a dose-dependent manner [15]. The risk of kidney cancer is significantly increased with higher SBP or DBP in a dose-dependent manner, even after adjusting for antihypertensive medication use. More frequent physician contacts in the population with HTN may contribute to the association of kidney cancer with HTN. The association of kidney cancer with HTN was also revealed in this study, with an HR of 1.58 (Table 3, model 2). However, the tendency for a higher association in the younger-aged population was not observed.

Insulin resistance and diabetes have been reported to be associated with various cancers. The chronic inflammatory state of insulin resistance and diabetes has been suggested as the pathophysiology. A retrospective cohort study in Taiwan using data from national health insurance (about one million subjects) and a meta-analysis including 24 studies reported that DM might increase the risk of kidney cancer [16,17]. In all subjects of the present study, DM increased the risk of kidney cancer with an HR of 1.21 (model 2). However, in the young-aged population, this association was not significant regardless of gender. A case-control study about kidney cancer and diet, including carbohydrate and fiber intake, has found that individuals with a high-glycemic diet and HTN have a 2.7 times higher risk of RCC [18].

Several studies have reported that obesity and weight gain during adolescence or childhood can increase the risk of RCC. A Swedish study using data from conscription assessment and the Swedish Cancer Registry has evaluated the influence of overweight and obesity during adolescence on RCC [19]. A unit increase in BMI was linked to a 6% increased risk of RCC. A large prospective US cohort study assessing BMI and the incidence of RCC has reported that weight gain in early (18–35 years) and mid- (35–50 years) adulthood is closely related to RCC, whereas weight gain after midlife (>50 years) is not [4]. In the present study, obesity and dyslipidemia (low HDL and high TG) showed a stronger association with kidney cancer in the young-aged population than in the old-aged population. In preclinical studies, lipidic metabolism was found to be involved in RCC. Considering the results of this study, a therapeutic application to the metabolic pathway could be considered [20].

The incidence of RCC in males was about twice as high as in females. Hormone effects might have contributed to such a gender difference. Estrogen-activated estrogen receptor ß (ERß) plays a tumor suppressive role [21,22,23]. ERß can reduce the downstream pathway of growth hormone and increase the apoptotic cascade. In our gender analysis, MetS, obesity, and central obesity showed higher associations with kidney cancer in young-aged males, while such an age difference was not significant in females. The tumor suppressive role of ERß might have provided a protective effect in young females.

This study has several limitations. Due to the characteristics of a longitudinal study, causal inference regarding MetS and kidney cancer was unavailable. There was no available information about the pathologic classification or disease burden of kidney cancer or cancer deaths. This cohort was mainly comprised of Korean participants. Thus, validation in other populations is required. Despite these limitations, this study is representative since it is a population-based study evaluating the association of MetS with kidney cancer according to age and gender.

Results of this study suggest that public health initiatives need to focus on the young-aged population for risk reduction of kidney cancer. Not only smoking and obesity but also MetS should be included in such initiatives.

## 5. Conclusions

Obesity and MetS were associated with kidney cancer. The association was stronger in younger populations than in older ones. In the male population, MetS, obesity, and central obesity showed higher associations with kidney cancer in the younger population, while there was no significant difference in such associations according to age in the female population.

## Figures and Tables

**Table 1:** Demographics of the study population divided by the presence of kidney cancer according to age.

Age (Years)	Total			20–39			40–64			≥65		
Kidney cancer	(−)	(+)	*p*	(−)	(+)	*p*	(−)	(+)	*p*	(−)	(+)	*p*
n	9,919,912	12,758		3,153,202	1297		5,500,258	7829		1,266,452	3632	
BMI (kg/m^2^)	23.71 ± 3.2	24.68 ± 3.09	<0.0001	23.13 ± 3.5	24.89 ± 3.68	<0.0001	24 ± 2.99	24.84 ± 2.96	<0.0001	23.9 ± 3.15	24.24 ± 3.11	<0.0001
WC (cm)	80.24 ± 9.06	84.61 ± 8.4	<0.0001	78.08 ± 9.7	83.41 ± 9.83	<0.0001	80.8 ± 8.54	84.47 ± 8.2	<0.0001	83.21 ± 8.38	85.35 ± 8.21	<0.0001
Glucose (mg/dL)	97.08 ± 22.83	102.27 ± 26.45	<0.0001	90.99 ± 16.01	93.66 ± 18.08	<0.0001	99.12 ± 24.34	102.79 ± 27.5	<0.0001	103.37 ± 26.82	104.24 ± 26.06	0.0507
SBP (mmHg)	122.41 ± 14.92	127.64 ± 15.4	<0.0001	118.48 ± 12.96	123.15 ± 14.38	<0.0001	122.87 ± 14.9	126.77 ± 15.06	<0.0001	130.16 ± 16.2	131.11 ± 15.82	0.0004
DBP (mmHg)	76.3 ± 9.97	78.9 ± 10.17	<0.0001	74.37 ± 9.28	77.46 ± 10.33	<0.0001	76.96 ± 10.15	79.33 ± 10.08	<0.0001	78.25 ± 10.05	78.48 ± 10.24	0.1789
HDL (mg/dL)	55.56 ± 20.98	52.65 ± 22.33	<0.0001	56.45 ± 18.09	53.07 ± 18.86	<0.0001	55.34 ± 20.88	52.74 ± 21.73	<0.0001	54.25 ± 27.1	52.31 ± 24.64	<0.0001
TG (mg/dL)	134.13 ± 92.77	151.31 ± 97.92	<0.0001	123.63 ± 92.77	154.21 ± 109	<0.0001	138.67 ± 94.47	154.07 ± 99.57	<0.0001	140.51 ± 82.57	144.32 ± 89.46	0.0054
Renal duration	8.26 ± 0.79	4.49 ± 2.48	<0.0001	8.31 ± 0.37	5.01 ± 2.32	<0.0001	8.3 ± 0.65	4.48 ± 2.49	<0.0001	7.92 ± 1.62	4.3 ± 2.47	<0.0001

BMI—body mass index; WC—waist circumference; SBP—systolic blood pressure; DBP—diastolic blood pressure; HDL—high-density lipoprotein; TG—triglyceride; SD—standard deviation. Data are presented as mean ± standard deviation.

**Table 2:** Risk of kidney cancer according to body mass index and waist circumference.

Age (Years)	Total			20–39			40–64			≥65		
Mets Var.	Incidence	Model 1	Model 2	Incidence	Model 1	Model 2	Incidence	Model 1	Model 2	Incidence	Model 1	Model 2
Body mass index (kg/m^2^)												
<18.5	0.07	0.67 (0.58, 0.76)	0.78 (0.68, 0.9)	0.02	0.73 (0.53, 1.02)	1.03 (0.74, 1.43)	0.09	0.77 (0.62, 0.96)	0.78 (0.62, 0.97)	0.29	0.9 (0.73, 1.1)	0.86 (0.7, 1.06)
18.5–22.9	0.11	1 (Ref.)	1 (Ref.)	0.03	1 (Ref.)	1 (Ref.)	0.12	1 (Ref.)	1 (Ref.)	0.33	1 (Ref.)	1 (Ref.)
23–24.9	0.17	1.55 (1.48, 1.62)	1.31 (1.25, 1.38)	0.05	1.72 (1.47, 2.02)	1.34 (1.14, 1.58)	0.17	1.44 (1.36, 1.53)	1.28 (1.2, 1.36)	0.38	1.17 (1.07, 1.27)	1.22 (1.1, 1.32)
25–29.9	0.21	1.94 (1.86, 2.03)	1.61 (1.54, 1.68)	0.09	2.85 (2.49, 3.25)	2.07 (1.8, 2.39)	0.23	1.88 (1.78, 2)	1.6 (1.52, 1.7)	0.39	1.19 (1.1, 1.29)	1.34 (1.24, 1.46)
≥30	0.22	2.07 (1.9, 2.25)	2.06 (1.89, 2.24)	0.10	3.25 (2.6, 4.06)	2.56 (2.05, 3.21)	0.28	2.31 (2.072, 2.57	2.25 (2.02, 2.5)	0.39	1.21 (1.0, 1.45)	1.66 (1.38, 1.99)
Waist circumference (cm, M/F)												
<70/<65	0.05	0.52 (0.45, 0.6)	0.7 (0.61, 0.81)	0.02	0.58 (0.43, 0.8)	0.8 (0.59, 1.1)	0.08	0.74 (0.61, 0.9)	0.81 (0.67, 0.99)	0.19	0.6 (0.44, 0.82)	0.54 (0.39, 0.74)
70–79/65–74	0.09	1 (Ref.)	1 (Ref.)	0.03	1 (Ref.)	1 (Ref.)	0.10	1 (Ref.)	1 (Ref.)	0.32	1 (Ref.)	1 (Ref.)
80–89/75–84	0.17	1.95 (1.86, 2.05)	1.44 (1.37, 1.51)	0.06	2.07 (1.81, 2.37)	1.58 (1.38, 1.82)	0.18	1.73 (1.63, 1.84)	1.39 (1.31, 1.48)	0.36	1.13 (1.03, 1.25)	1.2 (1.09, 1.32)
90–99/85–94	0.25	2.76 (2.62, 2.91)	1.81 (1.72, 1.91)	0.09	3.14 (2.67, 3.69)	2.28 (1.93, 2.7)	0.25	2.41 (2.25, 2.58)	1.8 (1.68, 1.93)	0.38	1.2 (1.08, 1.33)	1.37 (1.23, 1.51)
≥100/≥95	0.29	3.21 (2.94, 3.5)	2.27 (2.08, 2.47)	0.13	4.2 (3.25, 5.43)	3.35 (2.59, 4.34)	0.28	2.72 2.41, 3.06)	2.16 (1.92, 2.44)	0.45	1.42 (1.22, 1.65)	1.82 (1.57, 2.12)

**Table 3:** Risk of kidney cancer according to obesity and metabolic syndrome in different age groups.

Age (Years)	All		20–39		40–64		≥65	
	HR (95% CI)		HR (95% CI)		HR (95% Cl)		HR (95% CI)	
	Model 1	Model 2	Model 1	Model 2	Model1	Model2	Model1	Model2
Obesity	1.65 (1.6, 1.71)	1.48 (1.43, 1.53)	2.47 (2.21, 2.75)	1.89 (1.69, 2.12)	1.63 (1.56, 1.71)	1.48 (1.421.55)	1.12 (1.05, 1.2)	1.26 (1.18, 1.35)
Central obesity	1.93 (1.83, 1.98)	1.48 (1.43, 1.54)	2.38 (2.1, 2.69)	1.88 (1.65, 2.13)	1.72(1.64, 1.8)	1.47 (1.4, 1.54)	1.14 (1.07, 1.22)	1.28 (1.2, 1.37)
DM	1.7 (1.64, 1.76)	1.21 (1.17, 1.26)	1.43 (1.26, 1.62)	1.12 (0.98, 1.27)	1.44 (1.37, 1.5)	1.16 (1.11, 1.21)	1.16 (1.09, 1.24)	1.12 (1.04, 1.19)
HTN	2.47 (2.38, 2.56)	1.58 (1.52, 1.64)	1.97 (1.77, 2.2)	1.49 (1.33, 1.68)	1.97 (1.88, 2.06)	1.54 (1.47, 1.61)	1.41 (1.3, 1.53)	1.44 (1.33, 1.56)
low HDL	1.57 (1.51, 1.63)	1.38 (1.33, 1.44)	1.44 (1.27, 1.64)	1.38 (1.21, 1.57)	1.29 (1.24, 1.35)	1.34 (1.28, 1.4)	1.02 (0.96, 1.1)	1.32 (1.24, 1.41)
High TG	1.7 (1.64, 1.76)	1.29 (1.25, 1.34)	1.87 (1.67, 2.09)	1.29 (1.15, 1.45)	1.54 (1.47, 1.61)	1.25 (1.19, 1.3)	1.12 (1.05, 1.19)	1.22 (1.14, 1.3)
MetS	2.28 (2.2, 2.36)	1.56 (1.51, 1.62)	2.55 (2.25, 2.89)	1.82 (1.6, 2.07)	1.87 (1.78, 1.95)	1.5 (1.43, 1.57)	1.2 (1.13, 1.28)	1.37 (1.29, 1.47)
Number of MetS components								
1	1.67 (1.56, 1.77)	1.23 (1.16, 1.31)	1.48 (1.28, 1.72)	1.21 (1.04, 1.41)	1.36 (1.26, 1.47)	1.16 (1.07, 1.26)	1.09 (0.94, 1.27)	1.13 (0.97, 1.32)
2	2.5 (2.35, 2.66)	1.52 (1.43, 1.61)	2 (1.7, 2.35)	1.39 (1.18, 1.64)	1.88 (1.74, 2.03)	1.43 (1.33, 1.55)	1.22 (1.06, 1.42)	1.34 (1.16, 1.55)
3	3.21 (3.02, 3.42)	1.77 (1.66, 1.89)	3.11 (2.61, 3.7)	1.98 (1.65, 2.37)	2.26 (2.09, 2.44)	1.61 (1.49, 1.75)	1.27 (1.1, 1.47)	1.5 (1.29, 1.73)
4	4.29 (4.01, 4.58)	2.18 (2.04, 2.33)	4 (3.19, 5.01)	2.43 (1.93, 3.06)	2.97 (2.73, 3.23)	2.01 (1.85, 2.19)	1.44 (1.24, 1.67)	1.74 (1.51, 2.04)
5	5.25 (4.83, 5.71)	2.53 (2.33, 2.76)	4.98 (3.32, 7.48)	2.92 (1.94, 4.41)	3.67 (3.29, 4.09)	2.41 (2.16, 2.69)	1.53 (1.29, 1.81)	1.97 (1.66, 2.33)
Obesity/MetS								
(−/+)	2.32 (2.2, 2.44)	1.44 (1.37, 1.52)	2.3 (1.75, 3)	1.57 (1.2, 2.07)	1.71 (1.6, 1.83)	1.33 (1.24, 1.42)	1.14 (1.05, 1.24)	1.29 (1.19, 1.41)
(+/−)	1.42 (1.35, 1.49)	1.33 (1.26, 1.4)	2.18 (1.92, 2.49)	1.74 (1.52, 1.99)	1.41 (1.32, 1.5)	1.33 (1.24, 1.41)	0.99 (0.88, 1.12)	1.12 (0.99, 1.26)
(+/+)	2.61 (2.5, 2.73)	1.87 (1.79, 1.95)	3.47 (3.01, 4.01)	2.41 (2.08, 2.8)	2.27 (2.15, 2.4)	1.82 (1.72, 1.92)	1.26 (1.16, 1.37)	1.53 (1.4, 1.66)

HR—hazard ratio; CI—confidence interval; DM—diabetes mellitus; HTN—hypertension; HDL—high-density lipoprotein; TG—triglyceride; MetS—metabolic syndrome.

**Table 4:** Cutoff levels of body mass index and waist circumference in relation to kidney cancer risk.

Gender	Age (Years)	BMI Cutoff (kg/m^2^)	Youden’s Index	Sensitivity	Specificity	WC Cutoff (cm)	Youden’s Index	Sensitivity	Specificity
Total	20–39	23.9	0.229	0.608	0.621	81	0.244	0.645	0.598
	40–64	23.9	0.133	0.626	0.507	82	0.191	0.652	0.539
	≥65	23.5	0.058	0.609	0.449	84	0.106	0.592	0.514
Male	20–39	24.1	0.182	0.655	0.527	81	0.178	0.746	0.432
	40–64	24	0.106	0.643	0.463	85	0.121	0.601	0.521
	≥65	23.5	0.071	0.574	0.497	84	0.074	0.639	0.435
Female	20–39	21.6	0.117	0.496	0.621	72	0.097	0.462	0.635
	40–64	23.1	0.124	0.666	0.458	79	0.133	0.534	0.600
	≥65	24.4	0.095	0.567	0.528	84	0.076	0.496	0.580

BMI—body mass index; WC—waist circumference.

**Table 5:** Risk of kidney cancer according to gender, obesity, and metabolic syndrome.

Age (Years)	All		20–39		40–64		≥65	
	HR (95% CI)		HR (95% CI)		HR (95% CI)		HR (95% CI)	
Gender	Male	Female	Male	Female	Male	Female	Male	Female
Body mass index (kg/m^2^)								
<18.5	0.75 (0.64, 0.89)	0.7 (0.56, 0.89)	0.86 (0.49, 1.5)	1.01 (0.66, 1.53)	0.78 (0.59, 1.03)	0.76 (0.53, 1.11)	0.92 (0.73, 1.16)	0.69 (0.44, 1.09)
18.5–22.9	1 (Ref.)	1 (Ref.)	1 (Ref.)	1 (Ref.)	1 (Ref.)	1 (Ref.)	1 (Ref.)	1 (Ref.)
23–24.9	1.35 (1.28, 1.43)	1.29 (1.18, 1.41)	1.52 (1.27, 1.82)	0.74 (0.46, 1.19)	1.33 (1.24, 1.44)	1.2 (1.08, 1.34)	1.3 (1.08, 1.32)	1.27 (1.08, 1.47)
25–29.9	1.7 (1.61, 1.79)	1.53 (1.41, 1.66)	2.3 (1.96, 2.69)	1.27 (0.83, 1.92)	1.7 (1.59, 1.82)	1.45 (1.3, 1.6)	1.32 (1.2, 1.46)	1.39 (1.2, 1.61)
≥30	2.22 (2, 2.46)	2 (1.73, 2.31)	2.84 (2.23, 3.61)	1.77 (0.86, 3.61)	2.41 (2.11, 2.75)	2.04 (1.7, 2.44)	1.71 (1.31, 2.24)	1.64 (1.27, 2.12)
Waist circumference (cm)								
<70/<65	0.63 (0.52, 0.78)	0.74 (0.6, 0.92)	0.65 (0.38, 1.1)	0.82 (0.55, 1.22)	0.71 (0.54, 0.95)	0.91 (0.69, 1.21)	0.49 (0.34, 0.71)	0.74 (0.4, 1.37)
70–79/65–74	1 (Ref.)	1 (Ref.)	1 (Ref.)	1 (Ref.)	1 (Ref.)	1 (Ref.)	1 (Ref.)	1 (Ref.)
80–89/75–84	1.52 (1.43, 1.61)	1.42 (1.31, 1.54)	1.73 (1.48, 2.03)	1.15 (0.82, 1.61)	1.43 (1.32, 1.54)	1.37 (1.24, 1.52)	1.2 (1.08, 1.34)	1.19 (1, 1.43)
90–99/85–94	1.99 (1.87, 2.12)	1.62 (1.46, 1.78)	2.55 (2.13, 3.06)	1.06 (0.57, 1.96)	1.91 (1.75, 2.08)	1.62 (1.43, 1.83)	1.4 (1.24, 1.57)	1.32 (1.091.59)
≥100/≥95	2.61 (2.33, 2.9)	1.91 (1.64, 2.22)	3.79 (2.88, 5)	1.78 (0.73, 4.36)	2.51 (2.17, 2.89)	1.62 (1.3, 2.04)	1.83 (1.5, 2.23)	1.82 (1.44, 2.31)
MetS	1.64 (1.57, 1.71)	1.44 (1.34, 1.54)	1.82 (1.6, 2.09)	1.61 (0.91, 2.83)	1.55 (1.47, 1.63)	1.39 (1.27, 1.52)	1.42 (1.31, 1.53)	1.3 (1.15, 1.46)
Obesity	1.52 (1.46, 1.58)	1.45 (1.36, 1.56)	1.96 (1.74, 2.21)	1.41 (0.98, 2.03)	1.52 (1.44, 1.6)	1.41 (1.3, 1.54)	1.25 (1.15, 1.36)	1.29 (1.15, 1.45)
Central obesity	1.56 (1.49, 1.63)	1.35 (1.25, 1.45)	1.94 (1.7, 2.21)	1.21 (0.72, 2.01)	1.53 (1.44, 1.62)	1.33 (1.21, 1.47)	1.3 (1.2, 1.41)	1.25 (1.11, 1.4)
DM	1.21 (1.16, 1.26)	1.24 (1.15, 1.32)	1.08 (0.95, 1.24)	1.38 (0.96, 1.98)	1.14 (1.08, 1.2)	1.21 (1.11, 1.32)	1.11 (1.03, 1.21)	1.13 (1, 1.26)
HTN	1.61 (1.54, 1.68)	1.57 (1.46, 1.69)	1.52 (1.34, 1.71)	1.29 (0.87, 1.91)	1.55 (1.47, 1.64)	1.52 (1.39, 1.65)	1.45 (1.31, 1.6)	1.43 (1.24, 1.65)
Low HDL	1.43 (1.37, 1.49)	1.29 (1.21, 1.38)	1.39 (1.2, 1.6)	1.35 (0.99, 1.83)	1.4 (1.32, 1.48)	1.22 (1.12, 1.32)	1.36 (1.26, 1.48)	1.24 (1.1, 1.39)
High TG	1.33 (1.27, 1.38)	1.25 (1.17, 1.34)	1.27 (1.13, 1.44)	1.52 (1.01, 2.3)	1.29 (1.23, 1.36)	1.15 (1.05, 1.25)	1.25 (1.15, 1.35)	1.17 (1.05, 1.32)
Obesity and Metabolic syndrome								
MetS only	1.49 (1.4, 1.59)	1.39 (1.26, 1.53)	1.53 (1.15, 2.04)	2.38 (1.05, 5.39)	1.34 (1.23, 1.45)	1.32 (1.16, 1.51)	1.33 (1.3, 1.47)	1.23 (1.05, 1.44)
Obesity only	1.34 (1.26, 1.42)	1.43 (1.3, 1.57)	1.79 (1.55, 2.06)	1.47 (0.98, 2.19)	1.32 (1.23, 1.43)	1.36 (1.22, 1.53)	1.07 (0.92, 1.23)	1.22 (1, 1.5)
Both	1.97 (1.88, 2.08)	1.73 (1.59, 1.89)	2.49 (2.13, 2.91)	1.36 (0.63, 2.89)	1.9 (1.78, 2.02)	1.66 (1.48, 1.85)	1.54 (1.4, 1.71)	1.49 (1.29, 1.73)

HR—hazard ratio; CI—confidence interval; MetS—metabolic syndrome; DM—diabetes mellitus; HTN—hypertension; HDL—high-density lipoprotein; TG—triglyceride.

## Data Availability

The Korean National Health Insurance Corporation (NHIC) provides access to confidential data for researchers who meet certain criteria through their website. These are third-party data, and we did not have any special privileges. Anyone can submit a research proposal online (https://nhiss.nhis.or.kr/bd/ab/bdaba021eng.do, accessed on 10 November 2022) and, if approved by an NHIC evaluation committee, the researcher can obtain the de-identified NHIC dataset after paying a fee.
